# Supplementary material for: Dairy Farmers and Veterinarians’ Agreement on Communication in Udder Health Consulting
Source: Vet Sci. 2024 Dec 18;11(12):665. doi: 10.3390/vetsci11120665 (PMC11680391; doi:10.3390/vetsci11120665)
Supplement: Supplementary file 1 [file vetsci-11-00665-s001.zip › vetsci-3171500-supplementary.pdf]

Supplementary Material

Dairy Farmers and Veterinarians' Agreement on Communication in Udder Health Consulting

Michael Farre [1,*], Erik Rattenborg [2], Henk Hogeveen [3], Volker Krömker [1] and Carsten Thure Kirkeby [1]

[1] Department of Veterinary and Animal Sciences, Section for Production, Nutrition and Health, University of Copenhagen, 1870 Frederiksberg C, Denmark
[2] SEGES Innovation, Agro Food Park 15, 8200 Aarhus, Denmark
[3] Business Economics Group, Department of Social Sciences, Wageningen University and Research, 6706 KN Wageningen, The Netherlands
* Correspondence: mifa@seges.dk; Tel.: +45-23835400

Supplementary figure S1. The residuals of the BTSCC are aligned with the prediction of the model in a normal QQ plot.

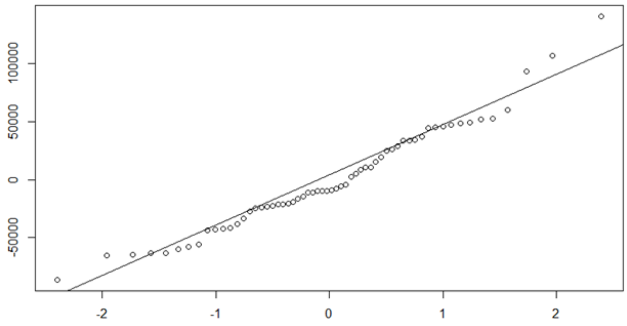

Supplementary Material

Dairy Farmers and Veterinarians' Agreement on Communication in Udder Health Consulting

Michael Farre [1,*], Erik Rattenborg [2], Henk Hogeveen [3], Volker Krömker [1] and Carsten Thure Kirkeby [1]

[1] Department of Veterinary and Animal Sciences, Section for Production, Nutrition and Health, University of Copenhagen, 1870 Frederiksberg C, Denmark
[2] SEGES Innovation, Agro Food Park 15, 8200 Aarhus, Denmark
[3] Business Economics Group, Department of Social Sciences, Wageningen University and Research, 6706 KN Wageningen, The Netherlands
* Correspondence: mifa@seges.dk; Tel.: +45-23835400

This table holds questions for the dairy farmer with question number, which construct the question is allocated, and the actual question to the dairy farmer. The questions have been translated from Danish. The subgroup with (-) indicates the question is not part of the analysis.

Table S1. The questions for the dairy farmer and the construct allocation.

Question number	Question to Construct #	Question asked to the dairy farmer
1	-	Who is answering the questionnaire at the farm visit?
2	-	Do visits always have an informal start with a social aspect?
3	-	If the answer to the previous question is yes – how much time do you spend on social talk?
4	-	Who typically sets the plan/goal (if there is one) for the meeting about udder health?
5	-	When you have a meeting about udder health, who is responsible for the agenda?
6	-	What situation led you to agree to the meeting?
7	2	How often do you personally initiate the discussions about udder health consulting in the herd?

8	2	In your opinion, is the frequency of follow-up sufficient to maintain a focus on the goals and critical control points (CCP)* in the herd?
9	2	How do you identify problems with udder health consulting in your herd?
10	2	If identifying problems through data, is the analysis done by your herd veterinarian?
11	-	If this is done based on data, does the herd veterinarian prepare an analysis?
12	-	How often does the herd veterinarian initiate the discussions about udder health consulting/mastitis in your herd?
13	2	To what extent do you agree that if there is a deviation from the (CCP)*, a joint decision is made about additional measures.
14	-	In your opinion, is the frequency of follow-up sufficient to maintain a focus on the goals and KPI in the herd?
15	1	To what extent do you agree that you are actively involved in discussing all the steps when discussing udder health consulting in your herd?
16	1	To what extent do you agree that there is an opportunity for fruitful discussion during the meetings with your veterinarian, with time for reflection?
17	1	To what extent do you agree that the herd veterinarian takes your questions seriously?
18	1	To what extent do you agree that you always receive full and satisfactory answers to your questions?
19	1	To what extent do you agree that meetings result in a written action plan with a clear division of responsibilities and tasks?
20	1	To what extent do you agree that action plans are always followed up within a specified time frame?
21	3	When the herd veterinarian suggests measures to improve udder health, how specific and precise are the recommendations?
22	3	Is the effectiveness of proposed measures evaluated very precisely?

23	3	To what extent do you agree that the proposed measures are always achievable?
24	3	To what extent do you agree that the proposed interventions are always realistic solutions to the problem in the herd?
25	3	To what extent do you agree that the proposed measures are always achievable within the specified time frame?
26	-	To what extent do you agree that the main reason for choosing a herd veterinarian is their knowledge of udder health consulting?
27	4	To what extent do you agree that the herd veterinarian always has in-depth knowledge of the udder health status in the herd?
28	4	To what extent do you agree that the herd veterinarian is an essential partner in managing udder health in the herd?
29	4	To what extent do you agree that the herd veterinarian is an important source of advice on udder health in your herd?
30	4	To what extent do you agree that measures to prevent mastitis in the herd are developed in close cooperation with the herd veterinarian?
31	4	To what extent do you agree that protocols, treatment, and vaccination plans are developed in close cooperation with the herd veterinarian?
32	4	To what extent do you agree that the herd veterinarian can turn scientific facts into practical and operational measures in the herd?
33	5	To what extent do you agree that good udder health consulting is of great economic importance for this herd?
34	5	To what extent do you agree that a disease like mastitis has a considerable impact on public opinion of milk production?
35	5	To what extent do you agree that mastitis has a significant influence on whether milk production can become CO2 neutral?
36	5	To what extent do you agree that mastitis is a severe welfare issue in the herd?
37	-	To what extent do you agree that clinical mastitis in cows requires excessive resources and time from the farm employees?

38	5	To what extent do you agree that the level of mastitis in the herd is a problem, bearing in mind consumers' attitudes and the sector's target of reducing antimicrobial consumption?
39	4	Do you consistently implement all the measures suggested by the herd veterinarian to improve udder health?
40	4	To what extent do you agree that the herd veterinarian's solutions for improving udder health in the herd are valuable, even if they are not consistently implemented?

Supplementary Material

Dairy Farmers and Veterinarians' Agreement on Communication in Udder Health Consulting

Michael Farre [1,*], Erik Rattenborg [2], Henk Hogeveen [3], Volker Krömker [1] and Carsten Thure Kirkeby [1]

[1] Department of Veterinary and Animal Sciences, Section for Production, Nutrition and Health, University of Copenhagen, 1870 Frederiksberg C, Denmark
[2] SEGES Innovation, Agro Food Park 15, 8200 Aarhus, Denmark
[3] Business Economics Group, Department of Social Sciences, Wageningen University and Research, 6706 KN Wageningen, The Netherlands
* Correspondence: mifa@seges.dk; Tel.: +45-23835400

Table S2. The questions for the herd veterinarian and the construct allocation.

Question number	Construct	Questions asked to the herd veterinarians
1	-	Who is answering the questionnaire?
2	-	Do visits always have an informal start with a social aspect?
3	-	If you answered "Yes" or "It depends" to the previous question – how much time do you spend on social talk?
4	-	Please think about the last time the topic of udder health consulting came up – what was the reason for this?
5	-	When you have a meeting with the farmer about udder health, who is responsible for the agenda?

6	-	Who led the last udder health meeting to facilitate the discussion and summarize conclusions?
7	1	To what extent do you agree that the farmer actively participates in the discussion regarding challenges with udder health?
8	1	To what extent do you agree that there is an opportunity for fruitful discussion between the farmer and herd veterinarian, with time for reflection during the meeting?
9	1	To what extent do you agree that the dairy farmer takes your questions about identifying problems in the herd seriously?
10	1	To what extent do you agree that you always receive full and satisfactory answers from the farmer when you try to troubleshoot the reason for poor udder health in the herd?
11	1	To what extent do you agree that udder health meetings result in a written action plan with a clear division of responsibilities and tasks?
12	1	To what extent do you agree that plans are always followed up within a specified time frame when working with udder health consultation?
13	2	How often do you personally initiate the discussions about udder health consulting in the herd?
14	2	In your opinion, is the frequency with which udder health is discussed sufficient to maintain a focus on the goals and (CCP)* in the herd?
15	2	How do you identify udder health consulting problems in the herd?
16	2	If this is done based on data, do you prepare an analysis and, in cooperation with the farmer, use this as a basis for deciding on the intervention?
17	-	If there is a significant deviation from the (CCP)*, how often is a joint decision about additional measures made?
18	2	If a (CCP)* exceeds the set target, are data analyzed further to identify the cause?
19	3	When you suggest measures to improve udder health, how specific and precise are your recommendations?

20	3	Is the effectiveness of the proposed measures evaluated very precisely?
21	3	To what extent do you agree that the proposed measures are always achievable in the herd?
22	3	To what extent do you agree that the proposed measures are always realistic solutions to the problem in the herd?
23	3	To what extent do you agree that the proposed measures are always achievable within your specified time frame?
24	4	To what extent do you agree that you have in-depth knowledge of the udder health status in the herd at all times?
25	4	To what extent do you consider yourself an essential partner in managing udder health in the herd?
26	4	To what extent do you agree that you provide essential advice on udder health in the herd?
27	4	To what extent do you agree that preventive measures are developed in close collaboration with the dairy farmer?
28	4	To what extent do you agree that protocols, treatment, and vaccination plans are developed in close collaboration with the dairy farmer?
29	4	To what extent do you agree that you successfully transform scientific data into applicable operational interventions in the herd?
30	4	Does the farmer consistently implement all the suggested measures to improve udder health?
31	4	To what extent do you agree that the solutions you suggest for improving udder health in the herd are always well received, even if they are not implemented?
32	5	To what extent do you agree that good udder health consulting is of great economic importance for this herd?
33	5	To what extent do you agree that diseases such as mastitis have a considerable impact on public opinion of milk production?

34	5	To what extent do you agree that mastitis has a significant influence on whether milk production can become CO2 neutral?
35	5	To what extent do you agree that mastitis is a severe welfare issue in this herd?
36	-	To what extent do you agree that clinical mastitis in cows requires excessive resources and time from the farm employees?
37	5	To what extent do you agree that mastitis is a serious problem in the herd, bearing in mind the sector's target of reducing antibiotic consumption?

*CCP – Critical Control Points (BTSCC, new infection rate lactation, recording clinical mastitis, Dry cow treatment).

Supplementary Material

Dairy Farmers and Veterinarians' Agreement on Communication in Udder Health Consulting

Michael Farre [1,*], Erik Rattenborg [2], Henk Hogeveen [3], Volker Krömker [1] and Carsten Thure Kirkeby [1]

[1] Department of Veterinary and Animal Sciences, Section for Production, Nutrition and Health, University of Copenhagen, 1870 Frederiksberg C, Denmark
[2] SEGES Innovation, Agro Food Park 15, 8200 Aarhus, Denmark
[3] Business Economics Group, Department of Social Sciences, Wageningen University and Research, 6706 KN Wageningen, The Netherlands
* Correspondence: mifa@seges.dk; Tel.: +45-23835400

Supplementary Table S3. Cohen's weighted kappa based on paired questionnaire responses from dairy farmers and herd veterinarians.

Cohen's weighted kappa	Question pair #	Question #; dairy farmer	Question #; veterinarian
0.09	1	15	7
-0.06	2	16	8
0.10	3	17	9
0.05	4	18	10
0.09	5	19	11
0.07	6	20	12

0.10	7	21	19
0.01	8	22	20
0.08	9	23	21
0.08	10	24	22
-0.05	11	25	23
0.05	12	27	24
0.01	13	28	25
-0.10	14	29	26
0.01	15	30	27
-0.01	16	31	28
0.10	17	32	29
0.09	18	33	32
0.10	19	34	33
0.06	20	35	34
-0.02	21	36	35
0.12	22	37	36
0.08	23	38	37
0.00	24	39	30
0.01	25	40	31
